# Establishment of Nature Reserves in Administrative Regions of Mainland China

**DOI:** 10.1371/journal.pone.0119650

**Published:** 2015-03-13

**Authors:** Ziliang Guo, Guofa Cui

**Affiliations:** School of Nature Conservation, Beijing Forestry University, Beijing, China; Chinese Academy of Sciences, CHINA

## Abstract

Nature reserves are widely considered as one available strategy for protecting biodiversity, which is threatened by habitat fragmentation, and wildlife extinction. The Chinese government has established a goal of protecting 15% of its land area by 2015. We quantitated the characteristics and distribution of nature reserves in mainland China and evaluated the expansion process for national nature reserves. National nature reserves occupy 64.15% of the total area of nature reserves. Steppe and meadow ecosystem, ocean and seacoast ecosystem, and wild plant nature reserves represent lower percentages, particularly in national nature reserves, in which they comprised 0.76%, 0.54%, and 0.69%, respectively, of the area. Furthermore, medium and small nature reserves compose 92.32% of all nature reserves. The land area under any legal protection has reached 14.80%, although only 9.78% is strictly protected. However, if 9 super-large national nature reserves, located in Southwest and Northwest China were removed, the percentage of strictly protected area decreases to 2.66% of the land area of China. The areas contained in nature reserves in each province are not proportional to the areas of the provinces, particularly for national nature reserves, with higher protection rates in Southwest and Northwest China than in other regions. Of the 31 provinces, 22 provinces feature strict protection of less than 4% of their areas by national nature reserves; these provinces are mainly located in East, Central, South, and North China. Moreover, the unevenness indexes of the distribution of nature reserves and national nature reserves are 0.39 and 0.58, respectively. The construction of nature reserves has entered a steady development stage after a period of rapid expansion in mainland China. In recent years, the total area of national nature reserves has increased slowly, while the total area of nature reserves has not increased, although the number of nature reserves continues increase.

## Introduction

Natural ecosystems destruction, wildlife extinction, and habitat fragmentation have increased seriously since the 19th century, due to rapid population growth, the expansion of human activities and the accelerating use of natural resources [1–6]. Global interest in biodiversity conservation has increased accordingly [7–9]. Protected areas (PAs) are one available strategy for protecting biodiversity and natural ecosystems [8], [10–13]. Since the establishment of Yellowstone National Park, the first modern PA, in 1872, PAs have been rapidly established and expanded in many countries [5], [8], [14], [15]. Protected areas covered approximately 13% of global land area by 2010 [8], [16]. Nevertheless, the 2010 biodiversity target to effectively preserve “at least 10% of each ecological region” is far from accomplished, resulting from the preference of PAs in high elevation, low productivity ecosystems, or biodiversity hotspots, etc [8], [16], [17].

There are many forms of PAs with many titles. The International Union for Conservation of Nature (IUCN) has developed a protected area management categories system, that includes strict nature reserve, wilderness area, national park, natural monument, habitat/species management area, protected landscape/seascape, and managed resource protected area [18]. However, the Chinese classification is very different from that of the IUCN. The formal protected areas are known as nature reserves (NRs) with specific restrictions on human activities in mainland China [8], [10], [19]. NRs are classified on the basis of their constructed administrative grades, administrative departments, and major protected objects [20], [21]. NRs are divided into the national NRs (NNRs), provincial NRs, municipal NRs and county NRs in mainland China, according to their constructed administrative ranks [20]. Compared with other levels of NRs, the establishment of NNRs is much stricter and must be approved by the State Council of China. In the aspect of the management, the exploitations and construction projects of NNRs must be permitted by the administrative departments of the State Council of China. In terms of their ecosystem characteristics and their major protected objects, NRs are also classified into 3 major categories, including natural ecosystem NRs, wildlife NRs and natural monument NRs, which are further subdivided into 9 types in mainland China (Table 1). Natural ecosystem NRs are divided into forest ecosystem NRs, inland wetland and water area ecosystem (inland wetland) NRs, steppe and meadow ecosystem (steppe and meadow) NRs, desert ecosystem NRs, ocean and seacoast ecosystem (ocean and seacoast) NRs. Wildlife NRs include wild animal NRs and wild plant NRs, while ancient organism remains NRs and geological formation NRs are types of natural monument NRs [20].

**Table 1:** Grade standard of NR sizes in mainland China.

Categories of NRs		Super-large(km^2^)	Extra-large(km^2^)	Large(km^2^)	Medium(km^2^)	Small(km^2^)
Natural ecosystem	Forest ecosystem	>10000	2000–10000	500–2000	100–500	<100
	Inland wetland	>10000	2000–10000	1000–2000	200–1000	<200
	Steppe and meadow	>10000	2000–10000	1000–2000	200–1000	<200
	Desert ecosystem	>20000	5000–20000	2000–5000	500–2000	<500
	Ocean and seacoast	>10000	2000–10000	1000–2000	200–1000	<200
Wildlife	Wild animal	>10000	2000–10000	1000–2000	200–1000	<200
	Wild plant	>10000	1000–10000	300–1000	50–300	<50
Natural monument	Ancient organism remains	>10000	1000–10000	300–1000	50–300	<50
	Geological formation	>10000	1000–10000	300–1000	50–300	<50

China is one of the 8 countries with the richest biodiversity in the world after Brazil, Indonesia, Colombia, Mexico, Australia, Madagascar and Philippines [22]. There are a large number of endemic species in China [22–24]. Global Biodiversity Conservation Priorities and Global Biodiversity Hotpots cover much of South China and Southwest China [1], [25–27]. Hence, the construction and management of the NR system of China has had a substantial influence on global biodiversity conservation. Since the establishment of NRs in 1956, the number and area of NRs has increased rapidly in mainland China [28–32]. Moreover, a short-term biodiversity target maintaining the area of territorial NRs at 15% of the land area and conserving 90% of the national key protected species by 2015 in mainland China was proposed [33]. How close are we to achieving this goal? How do the proportions of protected area vary among different regions and categories? Here, we present results on the structure of NRs and a comprehensive assessment of the areas protected within various administrative regions in mainland China.

## Materials and Methods

We collected data and established a database of NRs in mainland China, that includes area, type, protected object, date of establishment and other information. The database of the Ministry of Environmental Protection of the People’s Republic of China was used as our primary source for NRs (http://222.28.119.57/files/6083000000048A85/sts.mep.gov.cn/zrbhq/zrbhq/201309/P020130927386454626867.pdf). The data of NRs were primarily obtained from the NR administrations of all provinces. This data acquisition process began in 2013. After a promotion campaign, data on the distribution of all 2,671 NRs in mainland China were finally obtained. We also gathered the geographical boundary and attribute information of the existing 407 NNRs with higher conservation value and more representative ecosystems and wildlife than other levels of NRs from the Scientific Survey Report and General Plan of NRs. Data on NRs in Hong Kong, Macao and Taiwan were missing due to the difficulty of obtaining these data.

We analyzed the size of NRs and NNRs in mainland China, and established the grade standards of NR sizes based on the existing classification scheme in mainland China (Table 1).

In addition, we used Sigmplot10.0 and GIS10.0 to assess the distribution pattern of NRs and the expansion process of NNRs in mainland China. In this study, we developed an unevenness index of the spatial distribution of NRs (*E*) based on the Gini coefficient, which was proposed as a measure of inequality of income or wealth [34–36], as follows:

$$E=1-\frac{1}{n}(2\sum_{i=1}^{n-1}w_{i}+1)$$ (1)

where *w*_*i*_ is the cumulative protected ratio from region 1 to region I as a percentage of the total protected ratio in each region. The regions were ranked from small to large according to the protected ratio. The term *n* is the number of regions involved in the calculation. Theoretically, the unevenness index is between 0–1. The higher the unevenness index, the more uneven the spatial distribution of NRs. A value of 0.3 is usually used as the critical value of the unevenness index [34], [35]; for the NRs, *E* > 0.3, indicating relative district unevenness.

## Results

### NR levels

The number, area, and average area of NRs vary greatly among different levels (Table 2). The proportion of municipal and county NRs (53.46%) is greater than that of NNRs, whereas the total area of these NRs only represents 9.89% of all NRs. As of 2013, there are 407 NNRs with a total area (950,571.77 km^2^), accounting for 64.15% of the total area of all NRs (Table 2). Moreover, the average area of the NNRs is 2,335.56 km^2^, significantly higher than that of other NRs. Thus, NNRs seem to be more critical in biodiversity conservation than other levels of NRs in China.

**Table 2:** Statistics for NR levels in mainland China.

Levels	Number	Ratio of number(%)	Area(km^2^)	Ratio of area(%)	Average area(km^2^)
National	407	15.24	950571.77	64.15	2335.56
Provincial	836	30.30	384660.00	25.96	460.12
Municipal	406	15.20	43249.68	2.92	106.53
County	1022	38.26	103278.65	6.97	101.06
**Total**	2671	100.00	1481760.10	100.00	554.76

### NR types

In mainland China, areas covered by forest ecosystem, inland wetland, desert ecosystem and wild animal NRs, represent high percentages of the total area, at 21.19%, 19.45%, 27.62%, and 27.39%, respectively. However, the number and areas of other types of NRs are lower (Table 3). For instance, the areas covered by steppe and meadow, ocean and seacoast, and wild plant NRs are relatively smaller, accounting for less than 2% of the total NR area.

**Table 3:** Statistics for NR and NNR types in mainland China.

Types of NRs	NRs				NNRs			
	Number	Ratio of number(%)	Area(km^2^)	Ratio of area(%)	Number	Ratio of number(%)	Area(km^2^)	Ratio of area(%)
Forest ecosystem	1400	52.41	314040.72	21.19	191	46.93	148652.25	15.64
Inland wetland	335	12.54	288230.57	19.45	47	11.55	196930.12	20.72
Steppe and meadow	43	1.61	21582.65	1.46	3	0.74	7247.64	0.76
Desert ecosystem	33	1.24	409202.58	27.62	13	3.19	367001.78	38.62
Ocean and seacoast	74	2.77	7473.58	0.50	17	4.18	5095.20	0.54
Wild animal	523	19.58	405897	27.39	105	25.80	215706.81	22.70
Wild plant	141	5.28	18632.5	1.26	11	2.70	6532.35	0.69
Ancient organism remains	32	1.20	5503.86	0.37	7	1.72	1683.93	0.18
Geological formation	90	3.37	11196.2	0.76	13	3.19	1721.69	0.18
**Total**	2671	100.00	1481760	100.00	407	100.00	950371.77	100.00

Compared with NRs, the difference in areas covered by various types of NNRs is more notable. The areas covered by steppe and meadow, ocean and seacoast, and wild plant NNRs occupy a lower proportion of the total NNR area, less than 1%. By contrast, desert ecosystem and inland wetland NNRs represent 38.62% and 20.72% respectively, of the total NNR area (Table 3).

### NR sizes

In mainland China, the sizes of most NRs are less than 200 km^2^, and there are 1,732 small NRs, accounting for 64.84% of the total number of NRs (Table 4). Furthermore, large NRs only account for 5.13% of all NRs. Compared with desert ecosystem NRs, the number of large NRs of other types is lower, particularly for ocean and seacoast, and wild plant NRs. Among NNRs, the number of medium NNRs is the highest representing 54.05% of the total number of NNRs. There are 98 large, extra-large and super-large NNRs, accounted for 24.08% of the total number, but 92.58% of the total area protected by NNRs (Table 5).

**Table 4:** Statistics for NR sizes in mainland China.

Types of NRs	Super-large		Extra-large		Large		Medium		Small	
	Number	Area(km^2^)	Number	Area(km^2^)	Number	Area(km^2^)	Number	Area(km^2^)	Number	Area(km^2^)
Forest ecosystem	4	85131.81	13	52342.17	73	64932.80	413	85343.65	897	26290.29
Inland wetland	2	159250.19	11	40014.48	19	27259.86	107	48005.42	196	13700.62
Steppe and meadow	0	0.00	2	8370.00	5	6174.61	14	5214.59	22	1823.45
Desert ecosystem	2	343000.00	4	35950.59	4	16477.05	13	11802.70	10	1972.24
Ocean and seacoast	0	0.00	0	0.00	1	1530.00	7	3613.51	66	2330.07
Wild animal	9	243623.16	14	68457.92	19	27578.67	117	49646.54	364	16591.16
Wild plant	0	0.00	4	9484.88	6	2785.49	40	5135.59	91	1226.55
Ancient organism remains	0	0.00	1	1136.00	6	3448.06	5	543.03	20	376.77
Geological formation	0	0.00	2	6608.00	4	1629.84	18	2295.00	66	663.38
**Total**	17	831005.16	51	222364.04	137	151816.38	734	211600.03	1732	64974.53

**Table 5:** Statistics for NNR sizes in mainland China.

Types of NRs	Super-large		Extra-large		Large		Medium		Small	
	Number	Area(km^2^)	Number	Area(km^2^)	Number	Area(km^2^)	Number	Area(km^2^)	Number	Area(km^2^)
Forest ecosystem	2	53682.00	10	36274.11	31	29328.60	130	28406.65	18	960.89
Inland wetland	1	148252.23	7	24767.23	9	13112.56	24	10113.94	6	684.16
Steppe and meadow	0	0.00	1	5800.00	1	1367.94	0	0.00	1	79.70
Desert ecosystem	2	343000.00	1	8000.00	3	11586.94	5	4011.88	2	402.96
Ocean and seacoast	0	0.00	0	0.00	1	1530.00	7	2923.23	9	641.98
Wild animal	4	138736.30	9	42348.85	9	13164.97	45	18102.18	38	3354.50
Wild plant	0	0.00	1	4746.88	2	774.98	5	908.07	3	102.42
Ancient organism remains	0	0.00	0	0.00	3	1603.38	0	0.00	4	80.55
Geological formation	0	0.00	1	1008.00	0	0.00	4	638.33	8	75.36
**Total**	9	683670.53	30	122945.07	59	71469.37	220	65104.28	89	6182.52

Approximately 91.98% of the total area of NNRs is covered by only 39 extra-large and super-large NNRs in mainland China (Table 5). The distribution of extra-large and super-large NNRs is extremely uneven across the country. They are mainly distributed over Southwest China, Northwest China, and Northeast China. Of the 31 provinces assessed, 67.74% have no extra-large or super-large NNRs. The Super-large and extra-large NNRs in mainland China are listed in S1 Table.

### NR distribution in administrative regions

NRs are divided into terrestrial NRs and marine NRs. Terrestrial NRs cover an area of 1,421,158.40 km^2^ and formally protect 14.80% of the terrestrial area of mainland China. Moreover, there are 132 marine NRs, covering approximately 60,101.70 km^2^ in mainland China, including mangrove forests and seacoasts.

**Fig 1:**
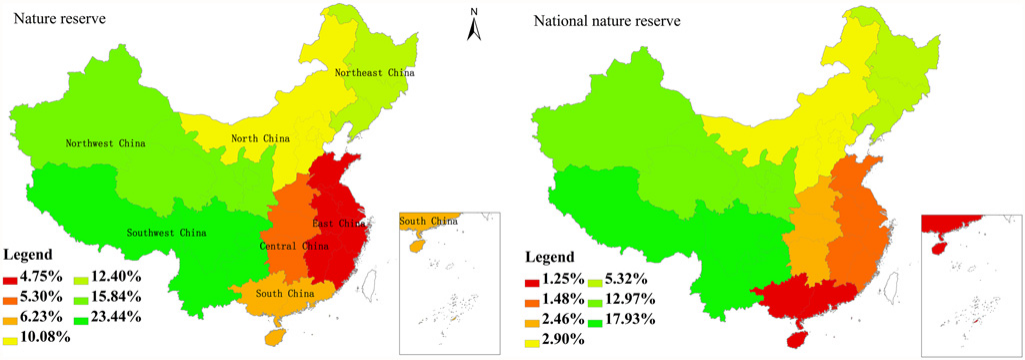
Proportion of land area covered by NRs and NNRs in different administrative regions.

NRs are geographically widespread in mainland China, but unevenly distributed in different administrative regions (Fig. 1). The percentages of protected area in East China (4.75%), Central China (5.30%), and South China (6.23%) are significantly lower than the national protection rate (14.8%). When considering only NNRs, less than 10% of the area is protected in most regions; Southwest China (17.93%) and South China (1.25%) are the most and least protected regions, respectively (Fig. 1).

**Fig 2:**
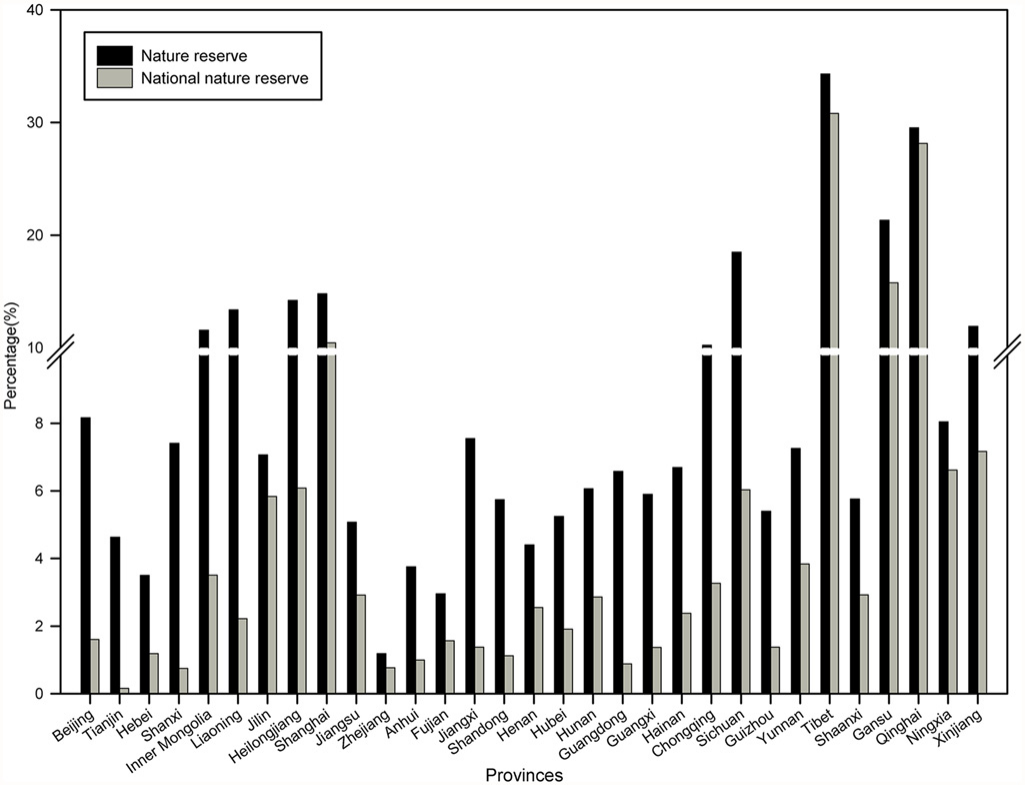
Proportion of land area covered by NRs and NNRs in different provinces.

Among the 31 provinces of mainland China, Tibet has the highest proportion ratio at 34.30%, closely followed by Qinghai at 29.53% (Fig. 2). However, the protection rates are less than 10% in 67.74% of the 31 provinces; these provinces are mainly located in East China, Central China, South China, and North China.

Taking NNRs into consideration only, the protection rates are less than 10% in most provinces with the exception of Tibet (30.79%), Qinghai (28.14%), Gansu (15.76%), and Shanghai (10.44%) (Fig. 2). Of the 31 provinces, 22 provinces strictly protect less than 4% of their areas in NNRs. Zhejiang and Anhui provinces in East China, Tianjin and Shanxi provinces in North China, and Guangdong province in South China have the least protection with less than 1% of their areas in NNRs.

**Fig 3:**
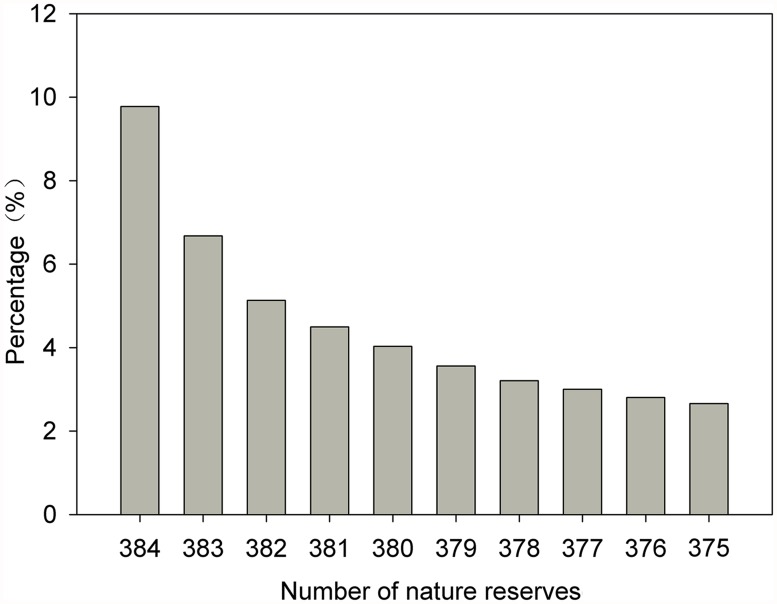
Accumulative percentages of land area protected by NNRs.

Super-large NNRs have great influence on assessing the total protected percentage of the land area. Hence, 9 super-large NNRs with areas greater than 10,000 km^2^ were removed in order from large to small, and the accumulative protected percentage of the land area was calculated (Fig. 3). The proportion of the national terrestrial area protected by NNRs declined from 9.78% to 2.26% in mainland China when the 9 super-large NNRs were excluded, revealing that 7.12% of the land area is covered by the 9 super-large NNRs. We used the unevenness index to evaluate the degree of unevenness of the distribution of NRs and NNRs in mainland China, which revealed that the distribution of NRs is uneven, with an unevenness index of 0.39, and that the distribution of NNRs is very biased, with an unevenness index of 0.58.

### Growth of the NNRs

The establishment of NRs began in the 1950s in mainland China, and the total number and area of NNRs were small and continued to increase slowly until the 1980s. By the end of 1985, only 15 NNRs had been established, and 0.10% of the national terrestrial area was formally protected by NNRs, covering an area of less than 10,000 km^2^ in mainland China.

**Fig 4:**
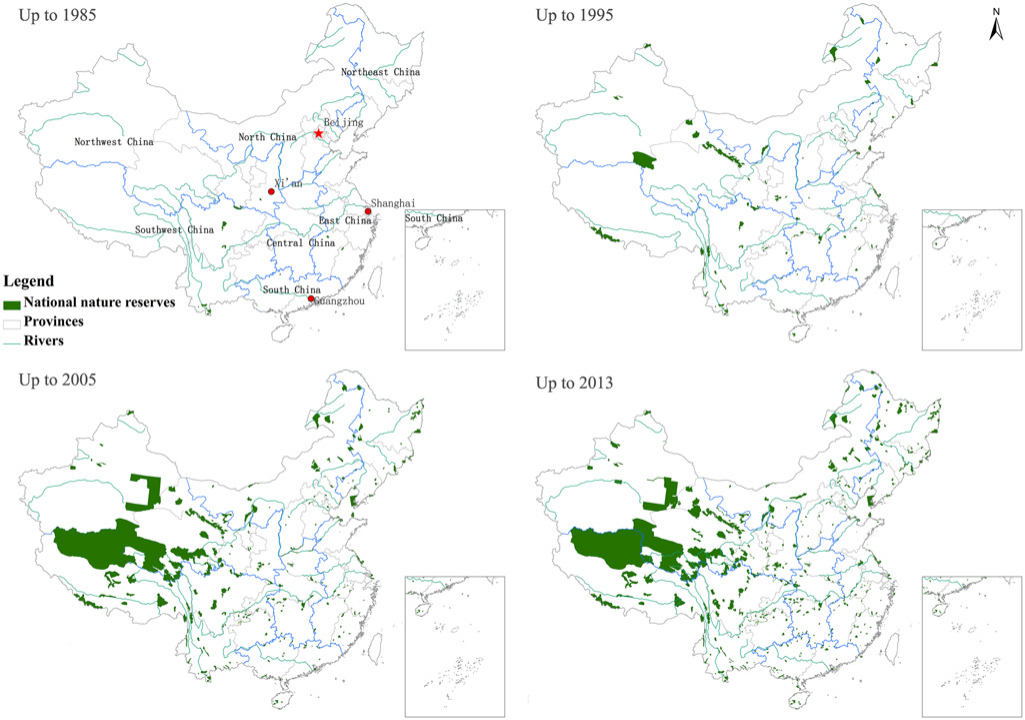
Changes in the spatial distribution of NNRs.

**Fig 5:**
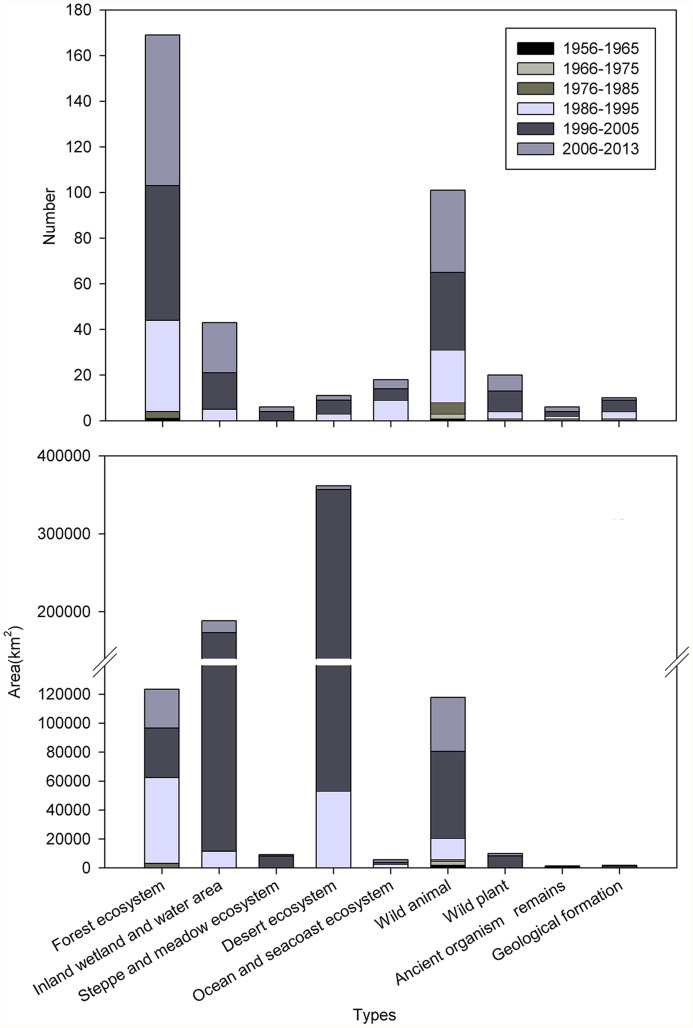
Increases in different types of NNRs.

Since 1984, the total number and area of NNRs has increased rapidly (Figs. 4 and 5). The number and area of NNRs grew faster in mainland China after the “National Wildlife Protection and Nature Reserve Construction” began in 2001. By 2005, 242 NNRs with approximately 730,000 km^2^ were protected, covering 7.6% of the land area in mainland China. Subsequently, the establishment of NNRs slowed, and the total area of NNRs gradually stabilized. All types of NNRs have continued to increase in number, although the timelines of their establishment have differed for the various types of NNRs. The area of inland wetland and desert ecosystem NNRs increased quickly at the end of the 20th century due to increasing concerns about wetlands and deserts. The construction of NNRs has been inconsistent in different regions and periods (Fig. 5). Many super-large and extra-large NNRs were established between 1996 and 2005 in Southwest China and Northwest China. The largest, the Tibet Qiangtang NNR (298,000 km^2^), is larger than most provinces in mainland China. However, the total area of NNRs is smaller in East China, South China and Central China, despite the presence of many NNRs in these regions.

**Fig 6:**
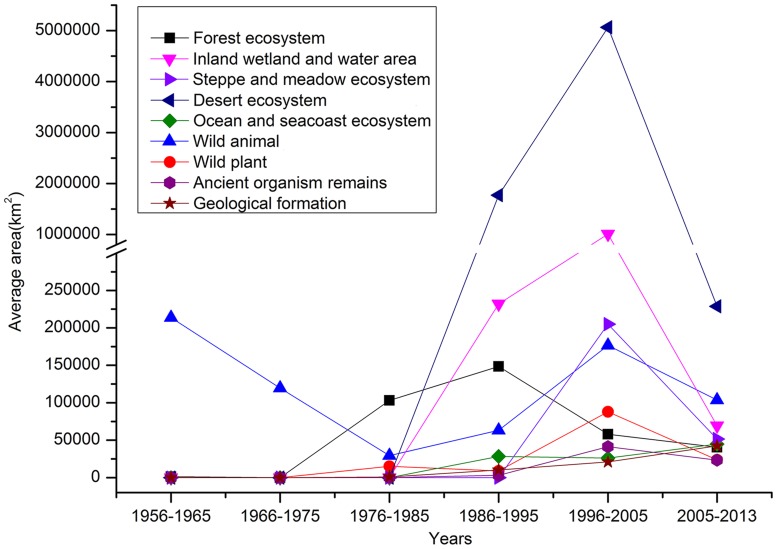
Average areas of different types of NNRs in different periods.

The mean area of all types of NNRs increased initially and then decreased over time, with the exception of ancient organism remains and geological formation NNRs (Fig. 6). The periods of the maximum mean area for each type of NNR differ. The period of the maximum mean area for forestry ecosystem NNRs occurred earlier than that of other NNR types.

## Discussion and Conclusion

NNRs occupied 64.15% of the total area protected by NRs, a greater proportion than that reported in previous analyses of mainland China due to an increase in NNRs and a decrease in NRs (Table 6) [28], [31], [37]. The total area of NRs and the size of each NR have a direct impact on the protection effectiveness for wildlife and their habitat [38]. Hence, NNRs could play more significant role in biodiversity and natural ecosystem conservation in mainland China. NNRs with administrative organization, adequate managers, and fixed funding sources for protection are considered as strict NRs and the main providers of biodiversity conservation in most parts of mainland China [39–41].

**Table 6:** Expansion of NRs and NNRs in mainland China in recent years.

Year of data	Number of NRs	Area of all NRs (thousand km^2^)	Number of NNRs	Area of all NNRs (thousand km^2^)
2003	1999	1439.80	226	887.13
2004	2194	1482.26	226	887.13
2005	2349	1499.50	243	889.90
2006	2395	1515.35	265	916.97
2008	2538	1489.43	303	912.03
2009	2541	1470.00	319	926.76
2010	2588	1494.40	319	926.76
2011	2640	1490.00	335	932.10
2012	2669	1487.28	363	941.05

In parts of China, the number and area of NRs are inadequate, and some types of NRs cannot adequately conserve their ecosystems, species, or other resources. There is still a lack of wild plant, steppe and meadow, ocean and seacoast NRs, particularly NNRs. For example, many rare and endangered wild plants have not been sufficiently protected [23], [24], although a large number of rare and endangered wild plants persist in mainland China [42], [43]. The number of NRs in each size category is also highly unbalanced. A small number of extra-large and super-large NRs, mainly distributed in Southwest China, Northwest China, and Northeast China with lower population density, represent more than 90% of the total area of NNRs. Whereas, in East China, Central China, and South China, most NNRs are medium and small NRs. Thus, some NRs with higher conservation value should be promoted as NNRs in these regions.

However, there is an unusual bias for the distribution of NRs in mainland China. In North China, East China, Central China and South China, the percentages of protected area are widespread lower, even less than 5%. Considering NNRs only, the proportion of protected area in the 13 provinces in East China, South China and North China, is lower than 2%. To ensure that NRs more effectively conserve biodiversity, the network of NRs should be constructed in concentrated zones of NRs in these regions. Subsequently, biological corridors could be also created among the NRs in the network, to enhance the ability of wildlife to adapt to climate change and human disturbances.

We determined that the unevenness index of the spatial distribution was 0.39 for NRs, and 0.58 for NNRs, both of which are higher than 0.3, also indicating that the distribution pattern of NRs and NNRs is extremely spatially unbalanced, particularly for NNRs. The unevenness index objectively reflects the degree of bias in protected areas. The geographical layout of NNRs should focus more on the provinces with lower protection ratios. We should promote the establishment of NRs and NNRs in Shanxi and Hebei provinces of North China, Zhejiang, Anhui, Fujian, Jiangxi and Shandong provinces of East China, Guangdong and Guangxi provinces of South China, and Guizhou province of Southwest China. Further analyses should be implemented in smaller scale, because the bias of protection could be more clear and specifical as the scale of analysis decreases [8].

The establishment of NNRs was inconsistent among different regions and periods for different types of NNRs. Although the increase in the total amount of NNRs was relatively constant from 1996 to 2013, the increase in the total area covered by NNRs slowed between 2006 and 2013, after the establishment of many super-large and extra-large NNRs. The area of NNRs has since increased extremely slowly, while that of NRs has not increased growth, although the number of NRs has continued to increase in recent years (Table 6) [44].

## Supporting Information

S1 Table Super-large and extra-large NNRs in mainland China. (DOC)
