# Supplementary material for: Establishment of Nature Reserves in Administrative Regions of Mainland China
Source: PLoS One. 2015 Mar 13;10(3):e0119650. doi: 10.1371/journal.pone.0119650 (PMC4358836; doi:10.1371/journal.pone.0119650)
Supplement: S1 Table — (DOC) [file pone.0119650.s001.doc]

Table S1: Super-large and extra-large NNRs in mainland China

| **Name** | **Area(km^2^)** | **Sizes** | **Types** | **Regions** |
| --- | --- | --- | --- | --- |
| Inner Mongolia Hui River | 3468.48 | Extra-large | Inland wetland | North China |
| Inner Mongolia Daqing Mountain | 3885.77 | Extra-large | Forest ecosystem | North China |
| Inner Mongolia West Erdos | 4746.88 | Extra-large | Wild plant | North China |
| Inner Mongolia Xilingol Grassland | 5800.00 | Extra-large | Steppe and meadow | North China |
| Inner Mongolia Dalai Lake | 7400.00 | Extra-large | Inland wetland | North China |
| Liaoning Dalian harbor seal | 6722.75 | Extra-large | Wild animal | Northeast China |
| Heilongjiang Wudalianchi | 1008.00 | Extra-large | Geological formation | Northeast China |
| Heilongjiang Zhalong | 2100.00 | Extra-large | Wild animal | Northeast China |
| Heilongjiang Dazhan river | 2116.18 | Extra-large | Inland wetland | Northeast China |
| Heilongjiang Xingkai Lake | 2224.88 | Extra-large | Inland wetland | Northeast China |
| Heilongjiang Nanweng River | 2295.23 | Extra-large | Inland wetland | Northeast China |
| Heilongjiang Raohe northeast black bee | 2700.00 | Extra-large | Wild animal | Northeast China |
| Jiangsu Yancheng Wetland | 2472.60 | Extra-large | Wild animal | East China |
| Sichuan Gongga Mountain | 4000.00 | Extra-large | Forest ecosystem | Southwest China |
| Sichuan Haizi Mountain | 4591.61 | Extra-large | Inland wetland | Southwest China |
| Sichuan Changshagongma | 6698.00 | Extra-large | Wild animal | Southwest China |
| Yunnan Xishuangbanna | 2417.76 | Extra-large | Forest ecosystem | Southwest China |
| Yunnan Baima Snow Mountain | 2821.06 | Extra-large | Forest ecosystem | Southwest China |
| Yunnan Gaoligong Mountain | 4052.00 | Extra-large | Forest ecosystem | Southwest China |
| Tibet middle reaches of Yarlung Zangbo River | 6143.50 | Extra-large | Wild animal | Southwest China |
| Tibet Yarlung Zangbo Grand Canyon | 9168.00 | Extra-large | Forest ecosystem | Southwest China |
| Tibet Siling Lake | 18936.30 | Super-large | Wild animal | Southwest China |
| Tibet Mount Qomolangma | 33810.00 | Super-large | Forest ecosystem | Southwest China |
| Tibet Qiangtang | 298000.00 | Super-large | Desert ecosystem | Southwest China |
| Gansu Gahai-zecha | 2474.31 | Extra-large | Forest ecosystem | Northwest China |
| Gansu Tao River | 2877.59 | Extra-large | Forest ecosystem | Northwest China |
| Gansu Annanba Bactrian Camel | 3960.00 | Extra-large | Wild animal | Northwest China |
| Gansu Dunhuang West Lake | 6600.00 | Extra-large | Wild animal | Northwest China |
| Gansu Anxi extremely arid desert | 8000.00 | Extra-large | Desert ecosystem | Northwest China |
| Gansu Yanchiwan | 13600.00 | Super-large | Wild animal | Northwest China |
| Gansu Qilian Mountains | 19872.00 | Super-large | Forest ecosystem | Northwest China |
| Qinghai Qinghai Lake | 4952.00 | Extra-large | Wild animal | Northwest China |
| Qinghai Kekexili | 45000.00 | Super-large | Wild animal | Northwest China |
| Qinghai Sanjiang source | 148252.23 | Super-large | Inland wetland | Northwest China |
| Xinjiang Kanas | 2201.62 | Extra large | Forest ecosystem | Northwest China |
| Xinjiang Tomur | 2376.00 | Extra large | Forest ecosystem | Northwest China |
| Xinjiang Aibi Lake | 2670.85 | Extra large | Inland wetland | Northwest China |
| Xinjiang Altun Mountain | 45000.00 | Super-large | Desert ecosystem | Northwest China |
| Xinjiang Lop Nor Bactrian Camel | 61200.00 | Super-large | Wild animal | Northwest China |
